# Levodopa-synergistic CBT intervention improves Parkinson’s disease with anxiety disorder by regulating the BDNF/PI3K/AKT pathway

**DOI:** 10.1007/s10072-025-07988-0

**Published:** 2025-02-12

**Authors:** Niu Ji, Shujin Lu, Bingchao Xu, Xinying Guan, Zhenping Xian, Deqin Geng, Dianshuai Gao

**Affiliations:** 1 Nanjing Medical University, No. 101, Longmian Avenue, Jiangning District, Nanjing, Jiangsu Province 211166 China; 2 The First People’s Hospital of Lianyungang, Lianyungang, Jiangsu Province 222000 China; 3 Department of Neurology, The Affiliated Hospital of Xuzhou Medical University, Huaihai West Road, Xuzhou, Jiangsu Province 221002 China; 4 School of Basic Medical Sciences, Xuzhou Medical University, No. 209, Tongshan Road, Xuzhou, Jiangsu Province 221004 China

**Keywords:** Parkinson's disease, Cognitive behavioral therapy, Anxiety disorder, BDNF, PI3K/Akt

## Abstract

**Background:**

Anxiety disorder is one of the most common and disabling neuropsychiatric syndromes in patients with Parkinson’s disease (PD), seriously affecting the quality of life and prognosis of PD patients.

**Objective:**

The objective of this study was to analyze the risk factors for anxiety in PD patients and to evaluate the effectiveness of cognitive behavioral therapy (CBT) in treating PD with anxiety disorder (PDAD).

**Methods:**

Baseline data were recorded for 211 PD patients and 139 PDAD patients, and multi-factorial and independent risk factors for anxiety disorder in PD patients were analyzed. ​The 139 PDAD patients were divided into clinical testing (CMO) and CBT groups. Assessments were taken at baseline and after the end of the intervention. A 5-month follow-up survey was conducted after the intervention. The mouse PD model was induced by MPTP, and the anxiety state of mice was detected by rotarod test and open-field test. The expression of BDNF/PI3K/Akt protein in serum and mouse brain was detected by western blot.

**Results:**

PDAD patients had significantly higher HAMA scores than PD patients. PSQI, ESS, HAMD, SCOPA-AUT, UPDRS-III and Hoehn-Yahr were independent risk factors for anxiety disorder in PD patients. After the intervention, the psychological state, cognitive function and quality of life improved in both the CMO and CBT groups, with the CBT group showing better improvement Results from follow-up showed that the number and frequency of falls was lower in the CBT group than in the CMO group, and that patients were more satisfied with the CBT intervention than the CMO group. L-dopa treatment alleviated anxiety in PD mice. L-dopa treatment increased BDNF, p-PI3K, and p-Akt protein levels. Moreover, the combination of L-dopa and CBT enhanced the boosting effect of L-dopa on these proteins.

**Conclusion:**

CBT is an effective treatment for anxiety in patients with Parkinson’s disease. Medications combined with CBT have been shown to be effective in improving depression, anxiety and quality of life in PDAD patients.

## Introduction

Parkinson’s disease (PD) is a common neurological degenerative disease in the elderly, affecting more than 6 million people worldwide [1, 2]. The survey showed that among the population aged 65 and above in China, there are 1700 cases of PD per 100,000 people, and the number of patients increases with the increase of age [3]. Although the specific pathogenesis of PD is still unclear in clinical practice, motor symptoms such as bradykinesia and muscle rigidity and non-motor symptoms such as dementia, anxiety and depression are the main characteristics of PD patients, which have a serious impact on the daily life of patients [4].

The incidence of anxiety disorder in PD patients can be as high as about 40%, and its incidence in PD patients is significantly higher than that in non-PD patients [5]. At the same time, as the most common non-motor symptom in PD patients, anxiety disorder may be the main or first clinical symptom indicating the occurrence of PD for some patients [6]. Loss of dopamine is also the basic cause of PD combined with anxiety disorder, which reduces mainly due to the lack of dopamine the inhibition on noradrenergic neurons, thus causing the generation of anxiety disorders [7]. Treatment with dopaminergic drugs can improve the motor symptoms of PD patients and significantly relieve anxiety symptoms, which further confirms that the generation of PDAD may be closely related to dopaminergic deficiency [8].

Cognitive behavioral therapy (CBT) refers to a class of science-based interventions aimed at directly manipulating dysfunctional thinking patterns and behavior patterns to reduce psychological distress [9]. CBT is the most commonly used anxiety psychotherapy in the general population [10] and can be used to improve obsessive-compulsive disorder, depression, and anxiety [11, 12]. Research showed that CBT has been shown to be effective in treating depression and impulse control disorders in PD [13, 14]. A recent randomized controlled trial found that CBT had a therapeutic effect on anxiety symptoms in PD patients [15]. However, the sample size of Moonen et al.’s study [15] is relatively small, which is not sufficient to prove the effect of CBT on anxiety in PD patients.

Therefore, this study expanded the sample size and took 350 patients with PD admitted to our hospital as research objects to explore the clinical effectiveness of CBT for anxiety in PD patients.

## Materials and methods

### Clinical cases and study design

In this retrospective cohort study, 350 PD patients admitted to our hospital from June 2019 to December 2022 were selected as the research objects. HAMA was used to investigate and diagnose PD patients with anxiety disorders. According to the survey results, they were divided into PD alone group (*n* = 211) and PD with anxiety disorder group (PDAD, *n* = 139). Patients with PDAD were randomly assigned to the control group (receiving clinical monitoring only (CMO)) and intervention group (receiving CBT plus clinical monitoring). All participants received standardized clinical, cognitive, and behavioral assessments, and Levodopa (L-dopa) was used as the anti-PD drug. In addition, participants underwent follow-up assessments within 5 months. During the follow-up period, the number of falls and injuries was recorded. The satisfaction of CBT intervention was investigated by oral inquiry, which was divided into satisfaction, basic satisfaction and dissatisfaction. The total satisfaction = (satisfied cases + basically satisfied cases) /total cases×100%. The scales used in this study are shown in Table 1.

PD diagnostic criteria: according to the relevant guideline criteria [16, 17], the diagnosis of PD must have three or more of the following characteristics: (1) resting tremor; (2) unilateral onset; (3) The disease showed a progressive trend; (4) Patients showed persistent asymmetric involvement after the onset of the disease; (5) with a clinical course of 10 years or more; (6) treatment with levodopamine for 5 years or more.

Inclusion criteria: (1) All enrolled subjects met the diagnostic criteria outlined in the Criteria for Parkinson’s Disease in China (2016 Edition) [36]; (2) Clinical data must be complete, with no omissions; (3) Subjects may be enrolled if they exhibit any of the following clinical symptoms: muscle rigidity, resting tremor at 4–6 Hz, or postural instability due to non-primary causes; (4) The initiation of voluntary movement is slow, and there is a significant reduction in movement. As the disease progresses, both the speed and amplitude of repetitive movements decrease; (5) Have not received any other psychiatric medication for treating anxiety.

Exclusion criteria: (1) Incomplete clinical data; (2) Secondary PD caused by craniocerebral trauma, cerebrovascular disease, encephalitis, or drugs; (3) Obvious cerebellar signs, cone system damage, and muscular atrophy; (4) Tumor, cirrhosis, heart failure, end-stage renal disease, or systemic acute infectious diseases; (5) History of mental illness or substance abuse, including alcohol or drug addiction; (6) Received nucleus destruction or deep brain stimulation; (7) No other concomitant psychiatric disorders; (8) Inability to cooperate with the treatment or irregular drugs medication adherence; (9) Death during the treatment period; (10) Refusal to provide their clinical data for this study.

The study is carried out in according with the Helsinki Declaration and approved by the medical ethics committees. The written informed consents was obtained from all participants.

**Table 1:** Assessment methods

Domain	Instrument
Anxiety disorder	Hamilton Anxiety Scale, HAMA [18]
Life quality	short form 36 questionnaire, SF-36 [19]
Sleeping quality	Pittsburgh Sleep Quality Index, PSQI [20]
Cognition impairment	Mini-Mental State Examination, MMSE [21] Montreal Cognitive Assessment Scale, MOCA [22]
Fatigue levels	Fatigue Scale-14, FS-14 [23]
Somnolence	Epworth Sleeping Scale, ESS [24]
Depression	Hamilton Depression Scale, HAMD [25]
PD grading	Hoehn-Yahr, H-Y [26]
PD progression	Unified Parkinson’ Disease Rating Scale, UPDRS [27]
Autonomic nervous function of the digestive system	the scales for outcomes in Parkinson ‘s disease-autonomic, SCOPA-AUT [28]
Balance ability	Berg Balance Scale, BBS [29]

### Sample collection

To collect blood samples, 5 mL of peripheral blood were collected from fasting patients between 8:00 and 9:00 in the morning. Subsequently, the blood samples were transferred into a tube without anticoagulants and centrifuge to obtain serum. The protein levels of BDNF, p-PI3K/PI3K, and p-AKT/AKT were detected by western blot.

### Animals and treatments

C57BL/6 mice (8 weeks old; 22–25 g) were housed with a 12-h light/dark cycle and the same sleeping condition. 1-Methyl-4-phenyl-1,2,3,6-tetrahydropyridine (MPTP) (Sigma-Aldrich) was intraperitoneally (i.p.) injected into the mice (15 mg/kg, dissolved in saline) for 5 consecutive days to induce PD. Sham mice were administered saline. The L-dopa (20 mg/kg, MedChemExpress, NJ, USA), was administered (i.p.) for 9 consecutive days after the last injection of MPTP.

### Behavioral test

For the rotarod test, mice were placed on an accelerated rotating apparatus and the speed increased from 4 RPM to 40 RPM in 5 min. Before the test, the mice were trained for three days. All mice were tested three times. The latency to fall was recorded and the data are expressed as the average of the three trials.

For open-field test, the mice were transferred to the laboratory 1 h before the test for adaptation. Next, the mice were placed in the middle of the cube box for 5 min under evenly distributed lighting. Use SMART video tracking software (SMART 3.0) to record the total movement distance in 5 min.

### Western blot

The total protein in serum and mouse brain tissues was extracted with RIPA buffer. BCA protein kit was used to evaluate protein concentration. Protein samples were separated by 10% SDS-PAGE and then transferred onto PVDF membranes. The membranes were incubated with primary antibodies at 4 °C overnight and cultured for 2 h with secondary antibody. The bands were visualized by ECL kit and analyzed by ImageJ software.

### Statistical analysis

All the data in this study was analyzed by SPSS 25.0 and graphed by GraphPad Prism 10.0. Normality and homogeneity of variance tests were used for quantitative data, independent sample t-tests were used for inter group data, paired sample t-tests were used for intra group data, and one-way ANOVA and F-tests were used for multi group data. The categorical variable data was subjected to χ² test; Pearson correlation coefficient analysis of the relationship between anxiety disorder and quality of life in PD patients; Univariate and multivariate analysis of risk factors for concurrent anxiety disorders in PD patients. *P* < 0.05 was considered statistically significant.

## Results

### Baseline data of PD patients and PD patients with anxiety disorder

The baseline data of PD patients and PDAD patients was shown in Table 2. In the PD group, there were 126 men (59.72%) and 85 women (40.28%), with an average age of (68.50 ± 5.42) years, a BMI of (22.43 ± 1.48) kg/m^2^, and a course of (10.21 ± 3.45) years. There were 144 cases of diabetes (68.25%), 167 cases of hypertension (79.15%), 98 cases of hyperlipidemia (46.45%), 81 cases (38.39%) of primary school education and below, 109 cases (51.66%) of middle school to high school education, and 21 cases (9.95%) of college education and above. In the PDAD group, there were 76 men (54.68%) and 63 women (45.32%), with an average age of (69.27 ± 5.98) years, a BMI of (22.38 ± 1.49) kg/m^2^, a course of (10.42 ± 3.35) years, 99 cases of diabetes (71.22%), 102 cases of hypertension (73.38%), 56 cases of hyperlipidemia (40.29%), 59 cases (42.45%) with primary school education or below, 69 cases (49.64%) from junior high school to senior high school, and 11 cases (7.91%) with college education or above. There was no significant difference in age, sex, BMI, course of disease, basic disease and educational level between PD patients and PDAD patients (all *P* > 0.05). The HAMA score of PDAD patients (23.50 ± 6.33) was significantly higher than that of PD patients (7.09 ± 3.74), the difference was statistically significant (*P* < 0.05).

**Table 2:** Comparison of baseline data between PD patients and PD patients with anxiety disorder

	PD (*n* = 211)	PDAD (*n* = 139)	t/x^2^	*P*
Age (years)	68.50 ± 5.42	69.27 ± 5.98	-1.249	0.212
Gender			0.872	0.350
Male	126 (59.72)	76 (54.68)		
Female	85 (40.28)	63 (45.32)		
BMI (kg/m^2^)	22.43 ± 1.48	22.38 ± 1.49	0.304	0.761
Illness duration (years)	10.21 ± 3.45	10.42 ± 3.35	-0.560	0.576
Diabetes (cases)	144 (68.25)	99 (71.22)	0.350	0.554
Hypertension (cases)	167 (79.15)	102 (73.38)	1.566	0.211
Hyperlipidemia (cases)	98 (46.45)	56 (40.29)	1.290	0.256
HAMA (score)	7.09 ± 3.74	23.50 ± 6.33	-30.438	< 0.001
Degree education			1.022	0.307
elementary school and below	81 (38.39)	59 (42.45)		
Secondary school	109 (51.66)	69 (49.64)		
University and above	21 (9.95)	11 (7.91)		

PD: Parkinson’s disease; PDAD: Parkinson’s disease with anxiety disorder; BMI: Body mass index; HAMA: Hamilton anxiety scale

### Related factors of anxiety disorder in PD patients

In the assessment of quality of life, the SF-36 score of PD patients (45.31 ± 9.31) was significantly higher than that of PDAD patients (29.47 ± 11.14) (t = 14.397, *P* < 0.001), indicating that anxiety disorder significantly reduced the quality of life of PD patients. In PD group, the score of PSQI, MMSE, MMSE, FS-14, HAMD, BBS, SCOPA-AUT, and UPDRS-III were (11.21 ± 3.11), (14.24 ± 8.45), (6.55 ± 2.40), (9.74 ± 2.35), (8.32 ± 2.33), (47.42 ± 3.60), (9.00 ± 1.37) and (20.09 ± 5.98). The H-Y stage was (2.03 ± 0.83), including 135 cases in the early stage and 76 cases in the middle stage. In PDAD group, the score of PSQI, MMSE, MMSE, FS-14, HAMD, BBS, SCOPA-AUT, and UPDRS-III were (14.91 ± 2.50), (13.46 ± 7.55), (9.35 ± 2.22), (10.05 ± 1.35), (19.25 ± 5.18), (47.58 ± 3.46), (12.23 ± 2.01) and (29.73 ± 3.66). The H-Y stage was (3.63 ± 1.07), including 26 cases in the early stage, 76 cases in the middle stage and 37 cases in the late stage. Univariate analysis showed that the PSQI, ESS, HAMD, SCOPA-AUT, UPDRS-III and H-Y of PD group were significantly lower than those in the PDAD group (all *P* < 0.05). There was no significant statistical difference between the two groups in MMSE, FS-14, and BBS scores (all *P* > 0.05), as shown in Table 3. Multivariate regression analysis in Table 4 showed that PSQI, ESS, HAMD, SCOPA-AUT, UPDRS-III and H-Y were independent risk factors for anxiety disorders in PD patients (all *P* < 0.05).

**Table 3:** Analysis of related factors of anxiety disorder in PD patients

	PD (*n* = 211)	PDAD (*n* = 139)	t/χ²	*P*
SF-36 (score)	45.31 ± 9.31	29.47 ± 11.14	14.397	< 0.001
PSQI (score)	11.21 ± 3.11	14.91 ± 2.50	-11.741	< 0.001
MMSE (score)	14.24 ± 8.45	13.46 ± 7.55	0.877	0.381
ESS (score)	6.55 ± 2.40	9.35 ± 2.22	-11.034	< 0.001
FS-14 (score)	9.74 ± 2.35	10.05 ± 1.35	-1.415	0.518
HAMD (score)	8.32 ± 2.33	19.25 ± 5.18	-26.805	< 0.001
BBS (score)	47.42 ± 3.60	47.58 ± 3.46	-0.409	0.683
SCOPA-AUT (score)	9.00 ± 1.37	12.23 ± 2.01	-17.927	< 0.001
UPDRS-III (score)	20.09 ± 5.98	29.73 ± 3.66	-17.018	< 0.001
H-Y (stage)	2.03 ± 0.83	3.63 ± 1.07	-15.617	< 0.001
early	135	26	100.22	< 0.001
middle	76	76		
late	0	37		

PD: Parkinson’s disease; PDAD: Parkinson’s disease with anxiety disorder; SF-36: short form 36 questionnaire; PSQI: Pittsburgh sleep quality index; MMSE: Mini-mental state examination; ESS: Epworth sleeping scale; FS-14: Fatigue scale-14; HAMD: Hamilton depression scale; BBS: Berg balance scale; SCOPA-AUT: the scales for outcomes in Parkinson’s disease-autonomic; UPDRS-III: Unified Parkinson’ disease rating scale-III; H-Y: Hoehn-Yahr

**Table 4:** Multivariate analysis of the independent risk factors of anxiety disorder in PD patients

	B	S.E.	Wald	*P*	OR	95%CI
PSQI	0.881	3.218	1.233	0.001	2.413	1.118–4.762
ESS	1.235	2.134	3.732	0.011	3.438	0.972–5.672
HAMD	2.155	1.295	2.244	0.019	8.628	3.571–11.247
SCOPA-AUT	1.872	3.356	3.991	0.021	6.501	2.215–9.994
UPDRS-III	1.997	2.341	3.271	0.008	7.367	3.446–10.721
H-Y	2.111	1.235	2.255	0.020	8.256	4.728–11.422

SF-36: Short form 36 questionnaire; PSQI: Pittsburgh sleep quality index; MMSE: Mini-mental state examination; ESS: Epworth sleeping scale; FS-14: Fatigue scale-14; HAMD: Hamilton depression scale; BBS: Berg balance scale; SCOPA-AUT: the scales for outcomes in Parkinson ‘s disease - autonomic; UPDRS-III: Unified Parkinson’ disease rating scale-III; H-Y: Hoehn-Yahr.

### Baseline data of PD patients with anxiety disorder in the CMO group and the CBT group

The baseline data is shown in Table 5. In the CMO group, there were 36 males (50.00%) and 36 females (50.00%) with an average age of (69.28 ± 6.21) years, a BMI of (22.43 ± 1.47) kg/m^2^, a course of (10.79 ± 3.41) years, 54 cases of diabetes (75.00%), 56 cases of hypertension (77.78%), 32 cases of hyperlipidemia (44.44%), 35 cases (48.61%) of primary school and below, 33 cases (45.83%) of high school to middle school, and 4 cases (5.56%) of college and above. Among them, there were 44 cases (61.11%) smoking, 45 cases (62.50%) drinking alcohol, 23 cases (31.94%) drinking coffee, 18 cases (25.00%) urinary incontinence, 17 cases (23.61%) constipation, 10 cases (13.89%) orthostatic hypotension, 18 cases (25.00%) regular exercise, 46 cases (63.89%) fluctuating movements, 24 cases (33.33%) dyskinesia, 56 cases (77.78%) frozen gait, LDE of (687.00 ± 205.67) mg, unilateral onset in 45 cases (62.50%), 27 cases (37.50%) had bilateral onset. In the CBT group, there were 40 men (59.70%) and 27 women (40.30%) with an average age of (69.27 ± 5.78) years, a BMI of (22.33 ± 1.53) kg/m^2^, a course of (10.02 ± 3.26) years, 45 cases of diabetes (67.16%), 46 cases of hypertension (68.66%), 24 cases of hyperlipidemia (35.82%), 24 cases (35.82%) with a cultural level of primary school or below, 36 cases (53.73%) from high school to middle school, and 7 cases (10.45%) from university or above. Among them, there were 34 cases (50.75%) smoking, 36 cases (53.73%) drinking alcohol, 20 cases (29.85%) drinking coffee, 14 cases (20.90%) urinary incontinence, 18 cases (26.87%) constipation, 9 cases (13.43%) orthostatic hypotension, 16 cases (23.88%) regular exercise, 45 cases (67.16%) motor fluctuations, 20 cases (29.85%) dyskinesia, 50 cases (74.63%) frozen gait, LDE of (688.55 ± 214.96) mg, unilateral onset in 42 cases (62.69%), bilateral onset in 25 cases (37.31%).

**Table 5:** Comparison of baseline data between CMO and CBT groups

	CMO (*n* = 72)	CBT (*n* = 67)	t/x^2^	*P*
Age (years)	69.28 ± 6.21	69.27 ± 5.78	0.009	0.993
Gender			1.318	0.251
Male	36 (50.00)	40 (59.70)		
Female	36 (50.00)	27 (40.30)		
BMI (kg/m^2^)	22.43 ± 1.47	22.33 ± 1.53	0.388	0.698
Illness duration (years)	10.79 ± 3.41	10.02 ± 3.26	0.548	0.173
Diabetes (cases)	54 (75.00)	45 (67.16)	1.040	0.308
Hypertension (cases)	56 (77.78)	46 (68.66)	1.478	0.224
Hyperlipidemia (cases)	32 (44.44)	24 (35.82)	1.073	0.300
Degree education			1.002	0.443
elementary school and below	35 (48.61)	24 (35.82)		
Secondary school	33 (45.83)	36 (53.73)		
University and above	4 (5.56)	7 (10.45)		
Smoke (cases)	44 (61.11)	34 (50.75)	1.514	0.219
Alcohol (cases)	45 (62.50)	36 (53.73)	1.097	0.295
Coffee (cases)	23 (31.94)	20 (29.85)	0.071	0.790
Urinary incontinence (cases)	18 (25.00)	14 (20.90)	0.330	0.566
Constipation (cases)	17 (23.61)	18 (26.87)	0.195	0.659
Orthostatic hypotension (cases)	10 (13.89)	9 (13.43)	0.006	0.938
Joint deformities (cases)	0 (0.00)	0 (0.00)	0.000	1.000
Regular exercise (cases)	18 (25.00)	16 (23.88)	0.024	0.878
Motor fluctuations (cases)	46 (63.89)	45 (67.16)	0.165	0.685
Dyskinesia (cases)	24 (33.33)	20 (29.85)	0.195	0.659
Freezing gait (cases)	56 (77.78)	50 (74.63)	0.190	0.663
LDE (mg)	687.00 ± 205.67	688.55 ± 214.96	-0.044	0.965
Side of onset			0.001	0.982
Unilateral	45 (62.50)	42 (62.69)		
Bilateral	27 (37.50)	25 (37.31)		

CMO: clinical monitoring only; CBT: cognitive behavior therapy plus clinical monitoring; BMI: Body Mass Index; LDE: Levodopa equivalents

### Effect of CBT intervention combined with L-Dopamine on Parkinson’s disease with anxiety disorder

**Fig. 1:**
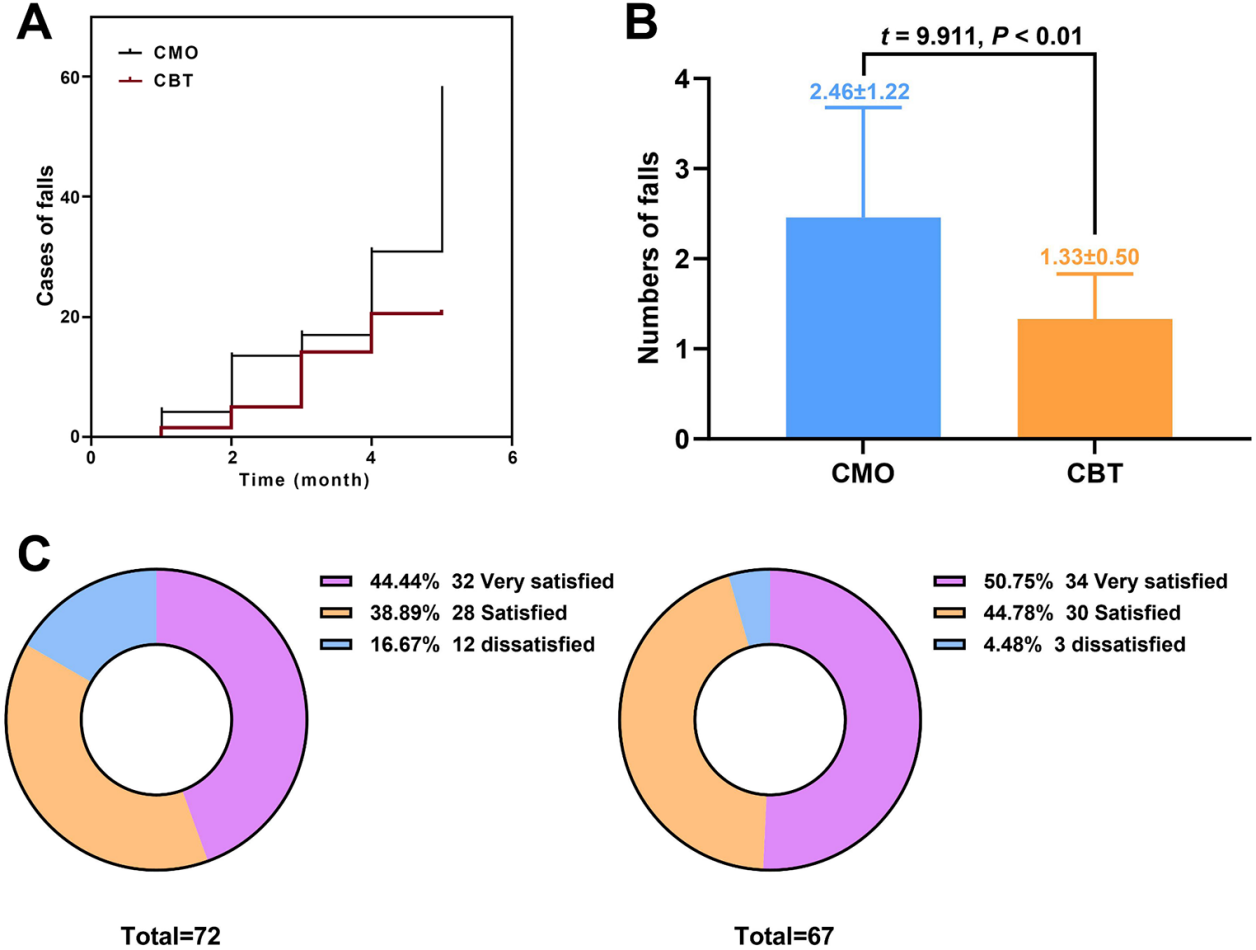
The results of follow-up survey. (**A**) Kaplan-Meier analysis of the number of falls in the two groups during the follow-up period; (**B**) The number of falls in both groups during follow-up; (**C**) Comparison of patients’ satisfaction with the intervention methods between the two groups

HAMA, HAMD, MMSE, MoCA, and SF-36 scores were used to evaluate the mental state, cognitive function and quality of life of PDAD patients in CMO group and CBT group before and after intervention, respectively (Table 6). There were no significant differences in HAMA, HAMD, MMSE, MoCA and SF-36 scores between the CMO and CBT groups before intervention. After intervention, HAMA and HAMD scores decreased significantly in both groups, while MMSE, MoCA and SF-36 scores increased significantly, with statistical differences (all *P* < 0.05). In CBT group, HAMA and HAMD scores decreased more, while MMSE, MoCA and SF-36 scores increased more significantly. These results indicate that CBT intervention can significantly reduce the adverse psychological state of PDAD patients and improve the cognitive function and quality of life of PDAD patients. In addition, we conducted a 5-month follow-up survey of PDAD patients. The survey included the number and frequency of falls and satisfaction with the intervention. During the follow-up period, the number and frequency of falls were significantly lower in the CBT group than in the CMO group (Fig. 1A and B). The results of oral inquiry on satisfaction showed that 60 patients in the 72 CMO group were satisfied with the intervention results (the sum of very satisfied and satisfied), and the overall satisfaction was 83.33%. Among the 67 patients in the CBT group, 64 were satisfied with the intervention results, with an overall satisfaction rate of 95.52%. Overall satisfaction with the intervention was significantly higher in the CBT group than in the CMO group (χ² = 5.356, *P* = 0.021) (Fig. 1C).

**Table 6:** The changes in mental state, cognitive function, and life quality of the two groups of PDAD patients before and after intervention

Groups	Intervention	HAMA	HAMD	MMSE	MoCA	SF-36
CMO (*n* = 72)	before	23.31 ± 6.16	19.29 ± 5.19	13.24 ± 7.39	11.85 ± 3.57	28.97 ± 11.77
	after	18.32 ± 3.47	15.68 ± 2.80	19.65 ± 5.39	18.42 ± 2.52	51.21 ± 10.90
	*t*	5.905	5.226	-6.406	-11.818	-12.402
	*P*	< 0.001	< 0.001	< 0.001	< 0.001	< 0.001
CBT (*n* = 67)	before	23.70 ± 6.55	19.19 ± 5.21	13.70 ± 7.78	10.88 ± 3.39	30.00 ± 10.49
	after	11.88 ± 3.90^*^	11.08 ± 3.37^*^	22.10 ± 4.50^*^	24.27 ± 3.67^*^	63.22 ± 12.80^*^
	*t*	12.159	10.354	-7.513	-12.098	-17.088
	*P*	< 0.001	< 0.001	< 0.001	< 0.001	< 0.001

CMO: Clinical monitoring only; CBT: Cognitive behavior therapy plus clinical monitoring; HAMA: Hamilton anxiety; HAMD: Hamilton depression; MMSE: Mini-mental state examination; MoCA: Montreal cognitive assessment; SF-36: Short form 36 questionnaire. *P < 0.05 vs. CMO group

### Levodopa-synergistic CBT intervention regulates the BDNF/PI3K/AKT pathway

**Fig. 2:**
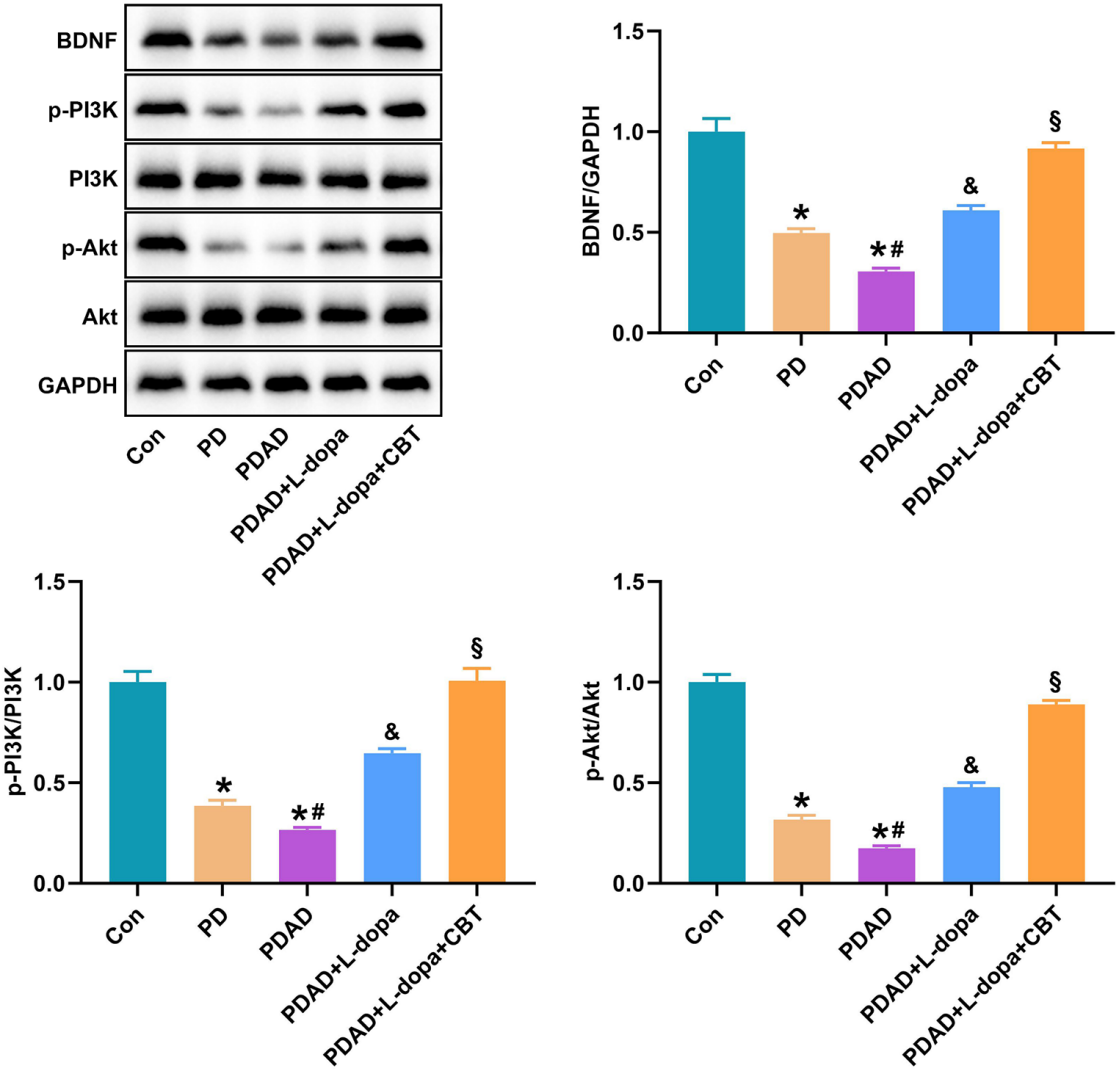
The protein expression of BDNF/PI3K/Akt pathway. *P < 0.05 vs. Con group, #*P* < 0.05 vs. PD group, &*P* < 0.05 vs. PDAD group, §*P* < 0.05 vs. PDAD + L-dopa group

**Fig. 3:**
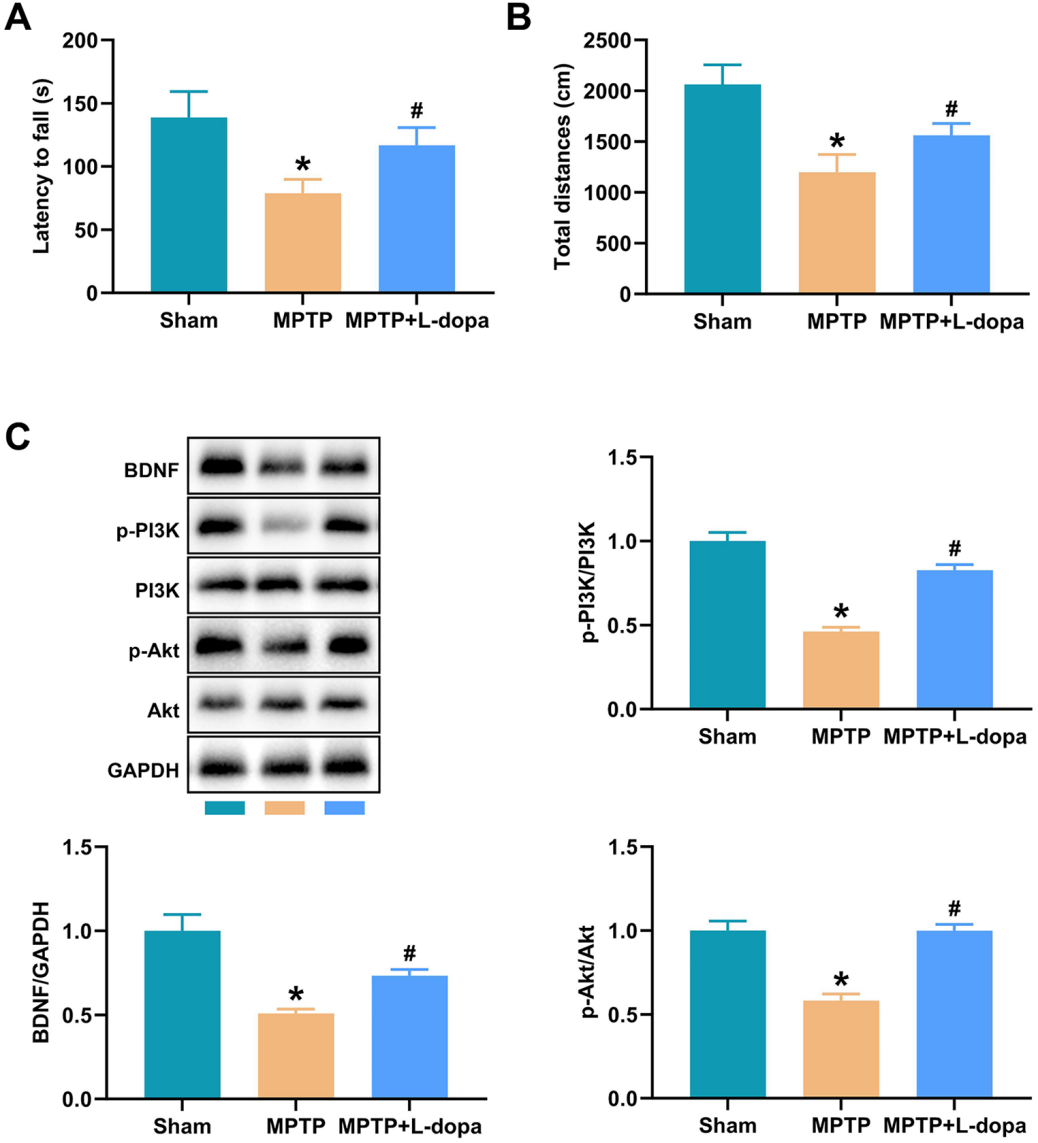
L-dopa regulates BDNF/PI3K/Akt pathway to alleviate PD. (**A**) The rotarod test of mice; (**B**) The open-field test of mice; (**C**) The protein expression of BDNF, p-PI3K/PI3K, and p-Akt/Akt. *P < 0.05 vs. Con group, #*P* < 0.05 vs. PD group

The protein expression of BDNF/PI3K/Akt in serum of patients was detected by western blot. The data was shown in Fig. 2. Compared with the Con group, the protein levels of BDNF, p-PI3K/PI3K, and p-Akt/Akt in PD and PDAD groups were markedly decreased, especially the PDAD group. L-dopa treatment enhanced the expression levels of these proteins and L-dopa + CBT further increased the expression of these proteins. Additionally, a mouse PD model was established by MPTP to verify the effect of L-dopa on PD. In the rotarod test, MPTP treatment significantly decreased the latency to fall. L-dopa significantly relieved the decreased latency to fall after MPTP treatment (Fig. 3A). In the open-field test, the MPTP group showed reduced movement compared with the Sham group (Fig. 3B). L-dopa obviously relieved the decrease of latency of falls and movement distances caused by MPTP (Fig. 3A and B). The western blot data showed that MPTP markedly decreased the protein levels of BDNF, p-PI3K/PI3K, and p-Akt/Akt (Fig. 3C). L-dopa eliminated the inhibitory effect of MPTP on expression of these proteins (Fig. 3C).

## Discussion

PD is a multiple disease with high incidence in recent years, with a markedly higher prevalence among males compared to females [30, 31]. This condition presents a spectrum of symptoms, encompassing both motor symptoms like bradykinesia, rest tremors, and rigidity, as well as non-motor symptoms such as cognitive impairment, sleep disturbances, and neurological disorders [31]. PD patients frequently grapple with depression, anxiety, and other detrimental emotional states, which exacerbate their health status and deteriorate their overall quality of life [32]. The prevalence of depression in PD patients is nearly 50% [33], and the prevalence of anxiety is 25-45% [34], significantly contributing to the elevated mortality rate among this patient population [35]. CBT, a crucial therapeutic approach in clinical practice, focuses on eradicating or mitigating negative emotions and empowering patients to self-manage their moods [36]. Despite its potential, there is a relative paucity of clinical research exploring CBT interventions specifically for PDAD. This study endeavors to investigate the impact of diverse intervention strategies on the mental state, cognitive function, and quality of life of PDAD patients.

Previous studies have suggested that the occurrence of PDAD is related to neurotransmitter abnormalities and amygdala volume changes [37, 38]. However, since anxiety disorder is relatively mild in the early stage of most patients, it has not attracted the attention of most doctors in clinical practice [39]. HAMA is a classic scale for evaluating anxiety in clinical neurosurgery, which can better reflect the severity of individual anxiety and can be used as an evaluation indicator to evaluate the clinical efficacy of intervention measures in nursing intervention and other treatment modalities [40]. In this study, HAMA score of DAD patients was significantly higher than that of PD patients. In addition, the quality of life survey results showed that the quality of life of PDAD patients was significantly lower than that of PD group. Pearson correlation analysis showed that there was a significant negative correlation between the degree of anxiety disorder and the quality of life, which was mainly because PD patients with severe anxiety had strong fear and discomfort in daily life, so they could not adapt to the social environment, and their accompanying symptoms such as panic, rapid breathing, trembling and weakness also seriously hindered daily activities. As a result, quality of life is reduced [41]. Univariate and multivariate analysis found that PSQI, ESS, HAMD, SCOPA-AUT, UPDRS-III and Hoehn-Yahr grades were correlated and independent risk factors for anxiety disorder in PD patients. The above analysis suggests that sleep quality, sleepiness, depression, PD severity, and autonomic nerve function of the digestive system may all contribute to the anxiety response in PD patients.

CBT is a mix of behavioral and cognitive interventions guided by applying scientific principles and is characterized by a problem-focused intervention strategy [42, 43]. Over the years, CBT has been used in the treatment of post-traumatic stress disorder, generalized anxiety disorder, obsessive-compulsive disorder, panic disorder, etc [44]. Its therapeutic purpose mainly focuses on guiding patients to change their negative thoughts, change negative behaviors by changing negative cognition, and further promote the clinical improvement of symptoms and the prognosis of the disease [45, 46]. In this study, both PD and PDAD patients were treated with L-dopa. The study found that after intervention with different intervention methods, the depression and anxiety scores of patients in both groups decreased, and the depression and anxiety scores of patients in the CBT group decreased more after intervention, indicating that CBT intervention can effectively reduce the degree of depression and anxiety of patients. In addition, the cognitive function of the two groups increased to varying degrees after the intervention, and the MMSE and MoCA scores of the CBT group were significantly higher than those of the CMO group after intervention. The quality of life survey results showed that the CBT group had significantly higher quality of life after the intervention compared to the CMO group. A 5-month follow-up showed that CBT significantly reduced the number and frequency of falls. And they were satisfied with the results of CBT. These results indicate that L-dopa combined with CBT is of great clinical significance in improving patients’ quality of life and disease prognosis.

Brain-derived neurotrophic factor (BDNF) belongs to neurotrophic factors, a family of proteins that support the function of the central nervous system and promote neuroprotection and nerve regeneration [47]. Neurodegenerative and neuropsychiatric disorders may be caused in part by an insufficient supply of neurons associated with BDNF and other neurotrophic factors [48]. Some studies have found the potential of BDNF as an indicator of CBT effect [49]. The serum levels of nerve growth factor were significantly higher in patients with generalized anxiety disorder after CBT [50]. A clinical study of panic disorder found that in patients receiving CBT, serum BDNF levels were significantly elevated in those who responded well [51]. Numerous studies demonstrated the association between BDNF and anxiety [52–54]. The study of Meng et al. demonstrated that PPM1F regulated anxiety-related behaviors by regulating BDNF expression [55]. Isochlorogenic acid B improved Pb-induced anxiety by modulating the BDNF signaling pathway [56]. Research by Lu et al. also showed that increasing the BDNF signaling pathway significantly improved anxiety in rats [57]. PI3K-AKT pathway is an intracellular signaling system, and the activation of this pathway may contribute to the prevention and treatment of PD [58, 59]. Studies have shown that BDNF can bind to tropomyosin receptor kinase B and activate the PI3K/Akt pathway, thereby avoiding the onset of Huntington’s disease [60]. Activation of the PI3K/Akt pathway promotes the survival and development of dopamine neurons [61]. Activation of PI3K/Akt pathway can reduce apoptosis in MPTP-induced PD animal models, thereby exerting neuroprotective effects [62]. In this study, we measured the expression of BDNF/PI3K/Akt protein in serum of each group. The results showed that BDNF, p-PI3K/PI3K and p-Akt/Akt were significantly down-regulated in PD and PDAD groups, while L-dopa treatment could up-regulate the expression of these proteins. Notably, the expression levels of BDNF, p-PI3K/PI3K, and p-Akt/Akt were significantly higher in the CBT intervention group compared to the L-dopa treatment group. The effect of L-dopa on PD was confirmed in mice. These results indicate that L-dopa can effectively improve PDAD, and L-dopa combined with CBT has a better effect.

## Conclusion

In conclusion, this study found that anxiety disorder in PD patients significantly reduced the quality of life of PD patients, and the independent risk factors for anxiety disorder were PSQI, ESS, HAMD, SCOPA-AUT, UPDRS-III and Hoehn-Yahr. L-dopa combined with CBT can reduce depression and anxiety and improve quality of life in PDAD patients. Therefore, L-dopa combined with CBT is recommended for clinical treatment of PDAD. The study has some limitations. We did not know the pre-treatment duration of participants’ PDAD episodes and the changes in BDNF levels among the subjects. Future research should also focus on analyzing the correlation between changes in BDNF levels and CBT treatment, as well as exploring additional non-pharmacological interventions to improve patients’ quality of life.

## Data Availability

The datasets used and analyzed during the current study are available from the corresponding author on reasonable request.
